# Challenges and Potential Solutions to Advance Global Cancer Drug Development

**DOI:** 10.1007/s43441-026-00936-w

**Published:** 2026-03-07

**Authors:** Axel Glasmacher, Kim Lyerly, Birgit Wolf, Pio Zapella, Lidia Zielinska, Emma Clark, Murielle Mauer, Bruno Paiva, Anja Schiel, Fergus Sweeney, Carin A. Uyl-de Groot, Marie von Lilienfeld-Toal, Jaap Verweij

**Affiliations:** 1 Department of Internal Medicine III, Universitätsklinikum Bonn, Venusberg-Campus 1, 53127 Bonn, Germany; 2 Cancer Drug Development Forum, Clos Chapelle-Aux-Champs 30, 1200 Brussels, Belgium; 3 Center for Applied Therapeutics, Duke University School of Medicine, 203 Research Drive, Medical Sciences Research Building, Suite 433,, Durham, NC 27710 USA; 4 Regulatory Policy & Science, Bayer AG, Research & Development Pharmaceuticals, Müllerstrasse 178, 13353 Berlin, Germany; 5 Debiopharm International S.A., Chemin Messidor 5, 1006 Lausanne, Switzerland; 6 Melanoma Patient Network Europe (MPNE), Fjallbo Selkna 152, 75597 Uppsala, Sweden; 7 Sarcoma - The Polish Sarcoma and Melanoma Patients Association, ul. Stefana Czarnieckiego 86/88 loc. 5, 01-541 Warsaw, Poland; 8 Product Development Data and Statistical Sciences, Roche Products Ltd, 6 Falcon Way, Shire Park, Welwyn Garden City, Hertfordshire, AL7 1TW UK; 9 Statistics Department, European Organisation for Research and Treatment of Cancer (EORTC) Headquarters, Av E. Mounier 83, 1200 Brussels, Belgium; 10 Clínica Universidad de Navarra, Cima Universidad de Navarra, Instituto de Investigación Sanitaria de Navarra (IDISNA), CIBER-ONC Numbers CB16/12/00369, Pamplona, Spain; 11 Regulatory and Pharmacoeconomic Statistics, Norwegian Medical Products Agency (NOMA: HTA and General Reimbursement), Grensesvingen 26, 0663 Oslo, Norway; 12 Erasmus School of Health Policy & Management, Institute for Medical Technology Assessment, Erasmus University Rotterdam, Bayle (J) Building, Room J8-37, Burgemeester Oudlaan 50, 3062 PA Rotterdam, The Netherlands; 13 Institute for Diversity Medicine, Ruhr-University Bochum, MA-Süd, Level 1, Room 52, 44780 Bochum, Germany

**Keywords:** Innovative trial design, Health technology assessment (HTA), Patient access, Patient diversity, Precision oncology, Patient-reported outcomes (PROs)

## Abstract

Despite recent advancements in oncology drug development, patient access to innovative cancer therapies remains inadequate. There is an urgent need for more patient-centric approaches, with meaningful patient input from trial design through to health technology assessment (HTA) consultation. Multi-stakeholder consensus calls for better representation of the diversity of the target population and integration of patients’ preferences in clinical cancer research by systematically collecting patient-reported outcomes using standardized methods, and acknowledging trade-offs between survival and long-term wellbeing. Furthermore, the generation of insufficiently robust data for regulatory and HTA decision-making continue to delay patient access to innovation. This could be mitigated through smarter study designs, including smaller, fit-for-purpose randomized studies and prospectively designed trials. Finally, concerted efforts are required to develop and validate novel intermediate/surrogate endpoints that enable earlier assessment of treatment outcomes to facilitate timely, evidence-based decisions that improve the patient experience across the cancer care continuum.

## Introduction

Successful drug development takes ~10–15 years from patent application to registration, with another 2–3 years of administrative procedures in the European Union (EU) including pricing, reimbursement negotiations and pharmacovigilance (Table 1) [1]. Whilst regulatory and health technology assessment (HTA) procedures are well-intended to safeguard society, persistent regional obstacles can inadvertently delay patient access to novel and effective precision oncology therapies, often arriving too late for patients with aggressive or relapsed cancers. The bureaucratic burden in the EU, including poor understanding of the recent EU In Vitro Diagnostic (IVD) Devices Regulation, has unduly delayed oncology drug development, particularly the initiation of early-phase and investigator-initiated trials. Accordingly, combined trials involving medicines and IVDs face complex regulatory hurdles and extended approval timelines, thereby hindering patient access to innovative cancer therapies.

**Table 1:** Stages of drug development, from early development to EU patient access

Stages	Description
1. Early development – Discovery & preclinical research	a. Identification of potential drug compounds b. Laboratory and animal studies to assess safety and efficacy
2. Clinical development	a. Phase 1: First-in-human trials to evaluate safety and dosage b. Phase 2: Studies to assess efficacy and side effects c. Phase 3: Large-scale trials to confirm efficacy and monitor adverse reactions
3. Regulatory submission	Marketing Authorization Application (MAA): a. Submission of comprehensive data to EMA b. Evaluation by the Committee for Medicinal Products for Human Use
4. EMA assessment: Review process*	a. Assessment of quality, safety and efficacy data b. Interaction with the applicant for clarifications
5. Approval across the EU	Centralized marketing authorization: If approved, the medicine receives authorization valid in all EU member states
6. Health technology assessment	National HTA bodies: a. Evaluation of the drug's added-value and cost-effectiveness b. Recommendations for reimbursement and pricing
7. Market access*	Negotiations between companies and HTA bodies & Reimbursement: a. Discussions with national health authorities b. Determination of pricing and inclusion in national formularies
8. Patient access	Availability: a. Drug becomes available to patients in EU countries† b. Ongoing pharmacovigilance to monitor safety in the real-world setting

EMA, European Medicines Agency; HTA, Health technology assessment

^*^Stages at which delays often occur

^†^Even if a drug becomes available, there can be issues with drug uptake, leading to delayed access

Based on the presentations at the conference by Carin Uyl-de Groot (Erasmus University Rotterdam, Netherlands)

Other EU-specific challenges include the stark between-country inequalities in patient access to new cancer therapies, with pronounced differences in authorization-to-reimbursement timelines and reimbursement affordability [2–4]. The complex EU regulatory environment has prompted the launch of initiatives promoting faster patient access to innovative therapies, such as the cross-sector COMBINE project aiming to streamline combined studies and harmonize regulatory frameworks [5], and the new HTA Regulation (HTAR; see Section "Marketing approval does not mean market (nor patient) access") [6]. The long and complex pathway of global oncology drug development is further complicated by an unmet need to generate evidence that is representative of the real-world target population and robust enough to facilitate efficient drug approval, placement on markets, reimbursement and patient access.

This paper reflects on multi-stakeholder discussions during the Cancer Drug Development Forum (CDDF) Annual Conference 2025 and provides some recommendations. A concerted effort from all stakeholders is urgently needed to accelerate the availability of life-saving innovative cancer therapies with improved toxicity profiles and quality of life (QoL) to patients in need.

## Smarter Study Designs to Shorten the Path to Patient Access

1. Single-arm trials (SATs) and challenges with external control data

Over recent years, both in the EU and the US, new drug approvals have increasingly been based on data from SATs, often justified by the difficulty to run phase 3 trials in small patient populations and/or the lack of effective existing treatments, and the use of non-time-dependent endpoints [7, 8]. Other features of SATs may include shorter trial duration, reduced costs, providing equitable treatment, and quicker patient access [8, 9]. However, benefit-risk assessments as performed by EU HTA processes require a comparison with the current therapeutic standard, therefore SATs using an external control arm (ECA) are not without their own challenges and could actually involve more work than designing a randomized trial from the get-go. For example, the use of complex analysis techniques associated with untestable assumptions (e.g., propensity score matching or causal inference) make any residual bias difficult to assess. Without randomization, one cannot control for unknown clinically important confounding or prognostic factors that may be discovered in future analysis. Accordingly, randomized controlled trials (RCTs) remain the ‘gold standard’ for data robustness in evidence-based medicine by controlling bias and allowing for time-dependent endpoints, such as progression-free survival (PFS) [10]. Although smaller randomized studies must be interpreted with caution (i.e., potential ‘false comfort zone’ – only larger sample sizes can truly balance for unknown confounders) and are not sufficiently powered to show statistically significant differences of time-to-event outcomes, these can improve clinical decision-making and time to approval when conducted early in drug development by offering better control against patient selection biases and more reliable safety and efficacy assessments.

Conversely, the additional collection of non-randomized data can complement and strengthen RCT results through assessing their external validity and powering subgroup analyses rather than replacing them. However, external data must be carefully selected and used in the right context in order to be relevant, i.e., distinguishing between any external data and true ECAs. Pre-planning and early engagement with decision-makers is absolutely required for collecting ECA data concurrently to the trial rather than as an after-thought once trial data is reported. In the era of precision oncology, with decreasing biomarker-positive histology-specific patient population sizes, histology-independent designs can increase sample size, and ECAs may still have to be considered in exceptional circumstances such as (ultra-) rare cancers. However, for precision oncology trials, greater rigor and proactivity is required to ensure the relevant mutation is expressed in external controls, information which might not be available in historical data [11]. Ultimately, prospectively collecting data outside of a randomized trial can truly strengthen evidence generation in both SATs and RCTs.

(2) Innovative trial designs to optimize evidence generation

There is an urgent need for more innovative trial designs, notably in disease/treatment settings in which there are small patient numbers. External borrowing approaches can sometimes be applied when there are insufficient controls to adequately power an RCT in particular disease settings [12]. For example, Bayesian dynamic borrowing can be used to select external controls from a contemporary, ongoing internal clinical trial to support an early analysis of phase 3 overall survival (OS) data in a treatment setting for which standard treatment has not evolved for decades (e.g. first-line diffuse large B-cell lymphoma). “Borrowing” patients from the control arm of another study may allow fewer ‘new’ patients to be treated with a well-established but inadequate control regimen; it also shortens the study time and enables more efficient trials by sharing control data between trials [13]. Nonetheless, the acceptability of such approaches from a regulatory perspective is not straight forward and very context-dependent.

**Fig. 1:**
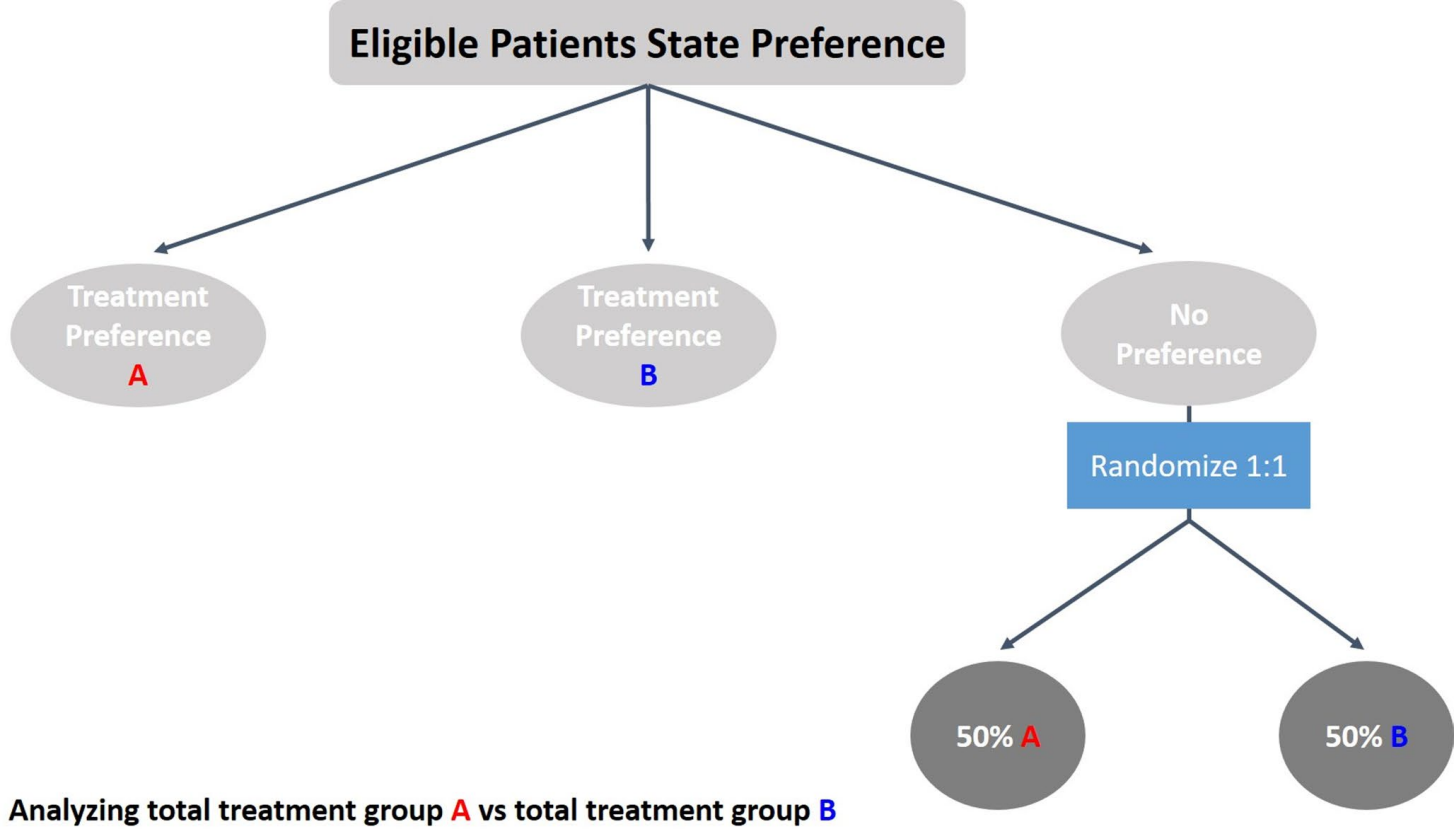
Partially randomized patient preference trial design.

Prospectively designed trials can generate more robust evidence, as well as build knowledge to address challenging public health questions regarding treatment optimization, usually conducted by academia/public sector (e.g., dose optimization, sequencing, multi-modality, de-escalation, or post-marketing authorization [MA]). Pragmatic approaches and elements in trials are currently implemented in specific situations (e.g., radiotherapy or surgery trials) when classical randomization is deemed more challenging. For example, the Trial within Cohorts (TwiCs) study design retains some features of a randomized study, using a large cohort from which some patients are selected for randomization as part of an experimental arm, with a multi-step informed consent applied only to relevant patients [14, 15]. Alternatively, partially randomized preference design (propensity score methodology) includes patients’ preference when randomization is not feasible (Fig. 1): patients can state their preference to undergo their treatment of choice, but in the absence of patient preference, they will be randomized between two treatment options [16] e.g., [17]. While they may improve patient recruitment and boost external validity, it is at the cost of potentially introducing bias (confounding) in the estimation of the effects of interest as patients’ or investigators’ pre-existing preferences for treatments may correlate with patients’ characteristics. Therefore, such studies including patient’s preference need to address selection bias using appropriate statistical methods like propensity score methodology [18]. Partially randomized preference design still uses randomization for a core group which should allow researchers to better quantify the actual “preference effect” by comparing the outcomes from the patients choosing versus being assigned. This implies that a sufficient number of patients actually accept to be randomized. Hence, when a classic RCT design is unfeasible, the use of mixed trial designs is a possible approach to maximize robust evidence generation while pragmatically collecting data from potential external controls [19]. However, when moving away from classical randomization, it is important to clearly address the statistical assumptions, estimands, and analytic implications.

Clinical trial methodology is one of the focus areas of Accelerating Clinical Trials in the EU (ACT EU), an important joint initiative of the European Commission, the European Medicines Agency (EMA) and Heads of Medicines Agencies established to create a favorable environment for research and development in life sciences through harmonization, innovation and collaboration with stakeholders. ACT EU also aims to improve ECAs, emphasize randomization in drug development, and move from multiple small studies investigating similar or overlapping questions (which often end up being under-powered) towards larger, more impactful and multi-state pragmatic trials, benefiting EU healthcare systems and patients in the process [https://accelerating-clinical-trials.europa.eu/].

(3) Patient perspectives in trial designs

An essential element for smarter trial designs involves incorporating patient perspectives from the very onset. It is counterintuitive to exclude the preferences of the very individuals the therapies aim to serve when designing studies intended to generate data for regulatory approval and reimbursement. Therefore, it would be logical to ask patients what matters most to them to ensure that trials generate evidence that is both clinically relevant and personally meaningful. At the same time, there must be a transparent and collaborative balance between generating ‘approvable’ data for regulatory bodies and addressing patient-prioritized outcomes. This balance should be achieved in partnership with patients, not in parallel to them. Going forward, multi-stakeholder collaboration is required to move beyond tokenism and systematically include patient insights from trial planning stages regarding treatment burden, practicalities and meaningful endpoints, through to market authorization and HTA decisions regarding reimbursement (see Sections "New endpoints to generate more pertinent evidence" and "Marketing approval does not mean market (nor patient) access"). Finally, addressing a risk of bias requires pre-planning at trial design stage for methodical data collection on all included patients regardless of their outcome, to maximize the data at hand and increase evidence robustness – every patient counts. One way forward could be to establish ‘patient steering committees’ or involve trained patient representatives to advise on strategies for maximizing data collection and minimizing attrition, particularly among under-represented or heavily burdened populations.

## Enhance Representation of Patient Diversity in Clinical Cancer Research

1. Addressing the lack of diversity

When a new drug is approved, it is released into a much broader and more heterogeneous population than that in which it was initially studied. Although RCTs are considered the gold standard, participants included in RCTs often do not represent the full range of actual patients treated in clinical practice, with many reports showing restrictive selection criteria in study protocols (e.g. [20–22]) and multiple examples of important differences between major subgroups in oncology clinical research and care (Table 2). Access to genomic testing may also vary widely in some regions, further exacerbating unrepresentative enrolment into precision medicine trials. Under-representation occurs when the prevalence of subpopulations affected by a given disease is not epidemiologically reflected in a respective trial population. There are also ‘badly’ represented groups in clinical trials, i.e., specific populations that may have been included but for whom data collection and analysis was not properly designed, or where data was collected but not well analyzed (e.g., sex or age bias).

**Table 2:** Bias in oncology care: Examples of significant differences in outcomes for major patient subgroups

Factors	Consequences
***Sex and/or age***	
Irinotecan plus chemo in 1L metastatic CRC – Efficacy by sex [49]	Treatment improved OS in men but worsened OS in women - The trial results initially reported an overall benefit of this treatment due to the majority of the trial population being male, thereby initially masking this detrimental effect in women
Bevacizumab plus chemo in 1L metastatic CRC – Efficacy by sex and age [50]	Although treatment improved OS in both sexes overall, subsequent analyses demonstrated no survival benefit in women aged < 60 years
Dosing of adjuvant chemo for colon cancer – Toxicity by sex [51]	Increased toxicity for women with colon cancer receiving adjuvant FU-based chemo
High-dose chemo plus ASCT in MM – Toxicity by sex [52]	Significantly higher rates of anemia and mucositis observed in women vs men
BSA-dosed cytotoxic drugs – Toxicity by size/body weight (and sex) [53]	Patients receiving the higher dose by body weight had very high rates of oral mucositis which corresponded to most female patients, while most of the males received a lower dose/body weight and experienced lower rates of this toxicity – demonstrating that we may be over-dosing smaller people and under-dosing larger people - In this case, sex was a potential surrogate for size (i.e. dosing by weight)
Sex-associated differences in AML-associated genetic alterations – Mutation patterns and prognostic impact by sex [54]	*SF3B1* mutations found to be male-specific adverse outcome prognosticators, and conversely, *WT1* was an adverse prognostic factor in women but not men
Sex-associated differences with genetic alterations in myelodysplastic syndromes – Mutation patterns and prognostic impact by sex [55]	*5q* deletion is more common in women than in men with myelodysplastic syndromes leading to better prognosis
***Ethnicity***	
Breast cancer survival outcomes by ethnic origin [56]	Poorer OS and a higher risk of breast cancer death in Black women vs White women for all tumor subtypes
Absolute neutrophil count by Duffy status [57] - Duffy null phenotype = clinically insignificant lower peripheral neutrophil count but adequate total body neutrophil count, most prevalent in people of sub-Saharan ancestry (80–100%) but also detected globally (< 1% in European descent). This phenotype is reported in 67% of African-Americans, therefore a significant proportion will be Duffy non-null with an ANC within the global reference range [57, 58]	- Exclusion from clinical trials based on ethnicity [57, 58] - Longer duration of treatment and resulting reduction in overall dose intensity in cancer therapy [59]

1L, first-line therapy; AML, acute myeloid leukemia; ASCT, autologous stem-cell transplantation; BSA, body surface area; chemo, chemotherapy; CRC, colorectal cancer; FU, fluoropyrimidine; MM, multiple myeloma; OS, overall survival

Based on the presentations at the conference by Marie von Lilienfeld-Toal (Bochum University, Germany) and Axel Glasmacher (University of Bonn, Germany & CDDF, Belgium)

A drug clinical development program should reflect the population that is meant to ultimately receive that treatment, and the lack of representative populations in clinical cancer research not only compromises the science but also the ethics of that research [23]. Accordingly, the external validation of results from RCTs is an urgent unmet need in oncology, resulting from design choices largely aiming at regulatory approval but translating into hurdles for HTA/payers/prescribers and patients. A major source of uncertainty in decision-making for all stakeholders downstream of regulators is the extrapolation of relative effectiveness for the entire patient population to be treated in the future. Diversity, equity and inclusion in clinical research has scientific and ethical foundations, not political, therefore better representation would not only ensure generalizability of the data but also the unbiased and fair practice of medicine for equal access [24, 25]. The recently launched CDDF Initiative ‘Diversity in Oncology Clinical Trials in Europe’, through a multi-stakeholder working group, aims to examine the parameters of trial inclusion, and to support the conduct of representative clinical cancer research and the applicability of trial outcomes to Europe’s diverse population [https://cddf.org/events/cddf-diversity-initiative/].

(2) Considerations for generating representative clinical data

With cancer drug development becoming increasingly multi-regional, all regulators face the issue that the majority of data submitted in MA applications across all therapeutic areas is generated in other regions. There are numerous examples of important differences in oncology care delivery between major subpopulations (e.g., between men and women and/or between different age groups; Table 2). Going forward, we must gather knowledge about target populations to set adequate inclusion/exclusion criteria for explanatory trials. We could systematically conduct disaggregated analyses of key subgroups, such as by sex and age at a minimum for each trial, and also analyze data from several trials to answer questions regarding smaller subpopulations.

Planning is essential (i.e., trial design, analysis, evaluation) to anticipate which populations are affected by the cancer and how that may impact the treatment benefit-risk. By asking the right questions upfront, subpopulation-specific differences in treatment effect (e.g. by sex, age or organ function deficiencies) should be revealed relatively early on in the drug development process. However, it remains necessary to balance the lack of data in specific populations with early access. Key considerations for designing trials with more representative populations involve a trade-off between sensitivity or internal validity (i.e. more narrowly defined study population) and generalizability or external validity (i.e. broadly defined, more representative real-world population) – first we must see if a drug has an effect, then determine which groups can most benefit (or not) from it. Finally, pragmatic trials with broader eligibility (i.e., fewer criteria) that welcome as many patients as possible can supplement clinical development programs to address the question of generalizability and provide external validity of explanatory or early-phase clinical trials, i.e. to ascertain treatment effectiveness in real-world clinical practice versus treatment efficacy in ‘ideal’/limited circumstances.

Industry and regulatory activities to achieve adequate representation of relevant subgroups in clinical trials are now well underway, but operational and political hurdles exist. When the audience at the CDDF 2025 Conference was polled regarding diversity, 70% of responding participants (N=78; 27% from regulatory agencies, 16% from patient advocacy groups, 13% from academic research institutions, and 45% from the pharmaceutical industry) were actively involved in diversity programs in clinical trials (Table 3).

**Table 3:** CDDF Conference audience voting on diversity

Questions	Poll Results
**Question 1: What are the most important objectives for diversity in clinical trials?**	N = 78*
Top 4 Responses (multiple answers were possible): 1. Generation of relevant information about special populations (e.g. teenagers, women of childbearing potential, elderly) 2. Well-designed, scientifically correct clinical trials with a clear selection and enrolment strategy for the target populations 3. Improvements of care for neglected/marginalized populations 4. Correct representation of affected populations in clinical trials	59% 49% 47% 41%
**Question 2: What are the most important problems for diversity in clinical trials?**	N = 78*
Top 4 Responses (multiple answers were possible): 1. Insufficient inclusion of representative populations 2. Inadequate planning for prospective analysis of subpopulations, and of data collection in some cases 3. Not sufficiently well defined data items to describe diverse populations 4. Insufficient knowledge about target cancer epidemiology, genotype, phenotype	71% 51% 36% 33%
**Question 3: Does your organization have a diversity plan for clinical trials?**	N = 63*
1. We currently have a diversity action plan for cancer clinical trials 2. We do not have a diversity action plan for cancer clinical trials 3. We do not have one, but are developing it	51% 33% 16%
**Question 4: Clinical trials diversity will be a clinical development priority – why?**	N = 65*
Top 4 Responses (multiple answers were possible): 1. Because of the scientific and clinical need for representative participations to demonstrate safety and efficacy of a new drug 2. Only because of regulatory requirements 3. Only if clinical development is made more efficient by increasing representative participation 4. Only after product marketing approval, e.g. if post-marketing studies are requested by regulatory authorities	65% 20% 6% 6%
**Question 5: Will a change in US FDA clinical diversity guidance impact your clinical development strategy?**	N = 60*
1. Not at all 2. We will wait and adjust to meet future US FDA guidance 3. We will adjust to meet EMA guidance 4. We will adjust to meet our own goals 5. We will deprioritize diversity/representative representation	40% 32% 13% 12% 3%
**Question 6: If the US FDA clinical trials diversity guidance is eliminated, how will your clinical development strategy change?**	N = 61*
1. We will meet EMA guidance 2. It will not change at all 3. It will meet our own guidance 4. It will be considered but not a priority	46% 25% 18% 11%

^*^Number of responders to individual poll questions

EMA, European Medicines Agency; US FDA, US Food and Drug Administration

The audience at the CDDF 2025 Annual Conference was polled on their views of the topic of diversity in cancer clinical trials. Twenty-seven percent of the answering participants (N = 78) were from regulatory agencies, 16% from patient advocacy groups, 13% from academic research institutions, and 45% from the pharmaceutical industry. Seventy percent of answering participants were actively involved in programs pertaining to diversity in clinical trials

## New Endpoints to Generate More Pertinent Evidence

1. Urgent need for intermediate endpoints in oncology

OS has long been considered the gold standard as the most clinically meaningful primary endpoint in oncology clinical trials. However, as prior successes of oncology drug development have significantly prolonged survival, its robust quantification requires time-consuming trials with large patient populations and long follow-up periods (sometimes >10 years), which can be affected by potential confounding from non-cancer deaths and the increasing availability of effective subsequent anti-cancer treatments [26, 27]. Alongside improved patient survival, the use of intermediate primary endpoints such as PFS has increased, with PFS now the most common primary endpoint in oncology RCTs and the basis for numerous EMA/US Food and Drug Administration (FDA) cancer drug approvals [27–29]. Furthermore, many key efficacy endpoints in cancer do not reflect patients’ experience, e.g. better quality of life, symptoms management, or need for surgical interventions (Table 4). Consequently, there is an urgent need to develop new validated oncology endpoints, including intermediate and surrogate endpoints to provide earlier read-outs of how patients are responding to a given therapy.

**Table 4:** CDDF Conference audience voting on endpoints

Questions	Poll Results
**Question 1: Which novel cancer drug development-related trial endpoints do you currently consider most relevant?** (*answers provided in a word cloud [not quantifiable] therefore listed in descending order of importance)*	N = 67* PFS > ctDNA > QoL, OS, EFS
**Question 2: Should PROs be used in every cancer drug development-related trial?** Yes No Not sure	N = 76* 61% 26% 13%
**Question 3: Is ctDNA quantification ready for use as cancer drug development-related trial endpoint?** No Not sure Yes	N = 79* 61% 29% 10%

^*^Number of responders to individual poll questions

ctDNA, circulating tumor DNA; EFS, event-free survival;. OS, overall survival; PFS, progression-free survival; PROs, patient-reported outcomes; QoL, quality of life

The audience at the CDDF 2025 Annual Conference was polled on their views on endpoints in cancer clinical trials

**Fig. 2:**
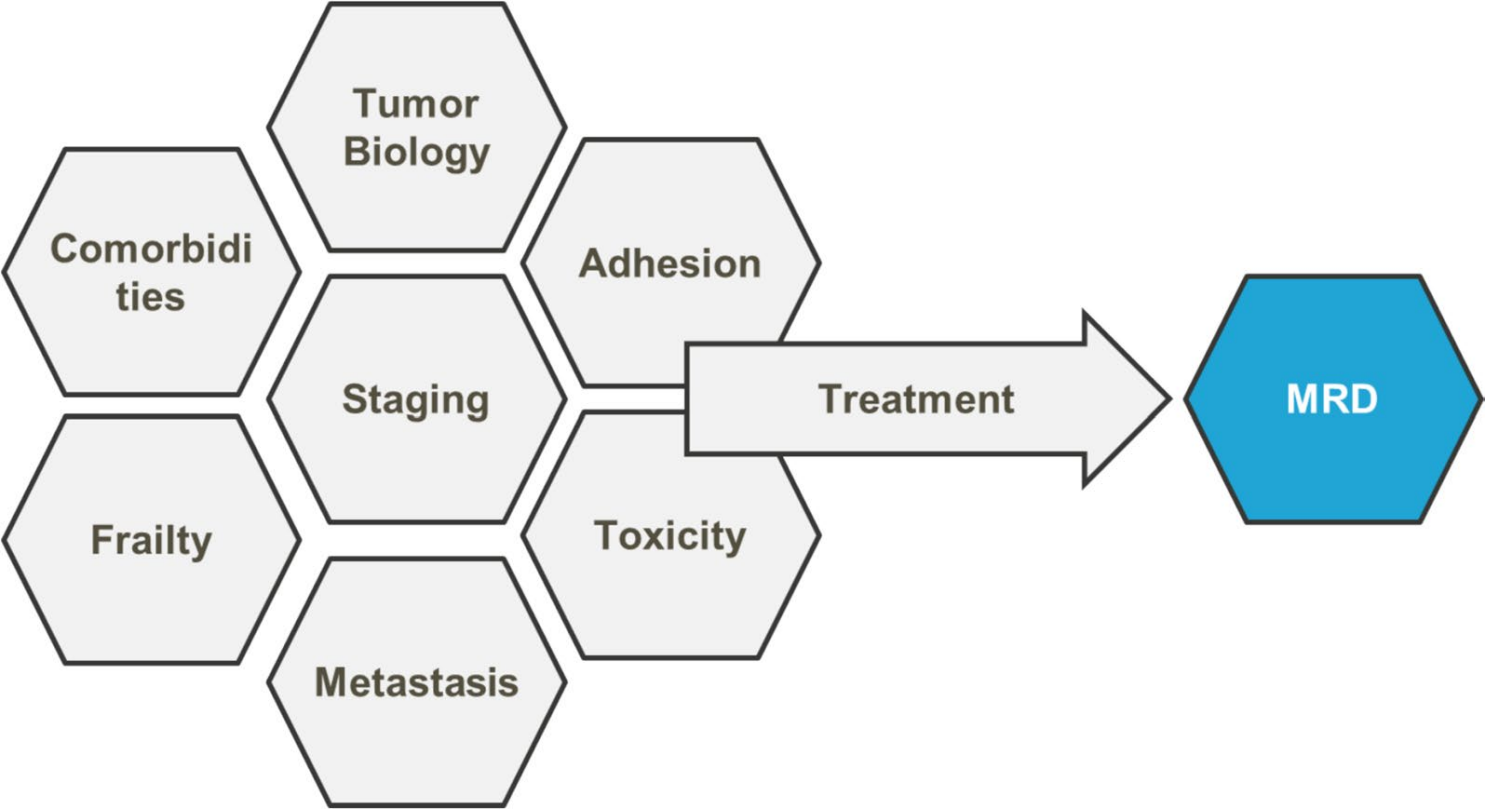
Minimal residual disease (MRD) as an earlier surrogate efficacy endpoint. Based on the presentation at the conference by Bruno Paiva (Clínica Universidad de Navarra, Spain)

**Fig. 3:**
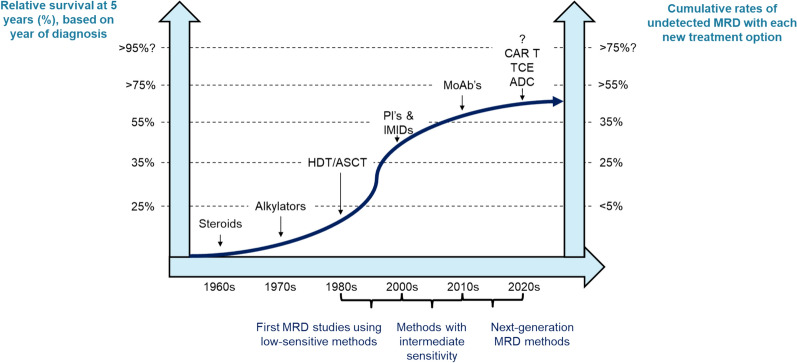
Deeper and durable MRD responses and prolonged survival in MM are interconnected. Adapted from Paiva B, et al. *Nat Rev Clin Oncol* 2025;22(6):424–438 [46-48], based on the presentation at the conference by Bruno Paiva (Clínica Universidad de Navarra, Spain). ADC, antibody–drug conjugate; CAR T, chimeric antigen receptor T-cell therapy; HDT/ASCT, high-dose therapy and autologous stem cell transplant; IMIDs, immunomodulatory drugs; MM, multiple myeloma; MRD, minimal residual disease; PIs, proteasome inhibitors; TCE, T-cell engaging bispecific antibodies

The recent FDA validation of minimal residual disease (MRD) as an intermediate endpoint in multiple myeloma (MM) sets an example for other cancer types [30]. With much progress in the treatment and survival of MM patients, PFS prolongation is not always a reliable surrogate of OS [e.g. 31], and increasingly longer periods are required for a documented PFS benefit or an observed lack of survival benefit (based on a usual MM trial design, ~7 years minimum for the interim PFS results and ~12 years for final PFS results – an unreasonable amount of time to solve a clear unmet medical need) [32]. MRD arose as a potential alternative early endpoint in MM as it captures multiple patient-related and tumor-related aspects in one single assessment (Figure 2). Numerous studies have consistently demonstrated a strong correlation between MRD negativity status and prolonged survival across different treatment settings in MM, and at both an individual patient-level and at trial-level (e.g. [32–38]). The interconnection between deeper and durable MRD responses and prolonged survival in MM can also be seen by relative 5-year survival with improved treatments over the years (from steroids, to transplant, new immunotherapies and so on) and progressively increasing rates of undetected MRD with each new treatment option, despite the use of ever more sensitive MRD methods/tools (Figure 3).

However, to validate MRD and circulating tumor DNA (ctDNA) as informative endpoints in drug development and patient care, it was necessary to address the respective limitations: the need to standardize assays and thresholds, the timepoints of measurements and especially, a statistically sound demonstration of the link between MRD and PFS/OS. Two hematological indications, MM and acute myeloid leukemia (AML), are the most advanced in meeting these requirements and have demonstrated the clinical value of MRD in large patient-level meta-analyses undertaken by comprehensive consortiums of academic study groups and pharmaceutical industry [39, 40].

In 2024, a consensus with the FDA was reached in MM on the use of MRD-negative complete response at ~9–12 months after treatment initiation as an intermediate endpoint for the accelerated approval of new treatments in MM (i.e. over ~3 years earlier than an expected interim PFS read-out) [38], demonstrating that a successful path to the validation and regulatory acceptance of new oncology endpoints is feasible. However, it was a costly and lengthy process (~10 years) requiring close multi-stakeholder collaboration between academic researchers, pharmaceutical industry and regulators, as well as an unprecedented sharing of data [41].

A similar approach for AML is pursued by the MRD Partnership and Alliance in AML Clinical Treatment Consortium (MPAACT) [39] and the Harmony Alliance in Europe [40]. The use of ctDNA is equally intensively researched and already successfully applied to guide treatment decisions as for the use of adjuvant therapies in muscle-invasive bladder cancer [42]. Also, the US-based non-profit-organization Friends of Cancer Research has started the ctMoniTR project to collect ctDNA data related to treatment response [https://friendsofcancerresearch.org/ctdna/]. Establishing robust new intermediate endpoints requires board public-private collaboration but will ultimately save time in drug development and optimize treatment regimens for patients.

(2) Patient-reported outcomes (PROs) and other patient experience data (PED)

Multi-stakeholder consensus on the urgent need to advance the use of patient preferences in clinical cancer research is clear, notably for the consistent collection of PROs and PED, including more accurate quantification of health-related QoL and improved analysis/reporting of PRO data (Table 4) [43]. Whilst clinical outcomes are methodically collected and reported whether favorable or not, PRO or QoL outcomes are often not collected when treatments fail (as progressing patients usually leave the study thereby making further QoL measurements unfeasible, leading to a severe bias in all reported PROs, or ‘survival bias’). Similarly, QoL that has not deteriorated will be interpreted as a treatment benefit, despite the absence of any comparator baseline QoL data.

Regulatory decision-making considers the totality of evidence, requiring complex trade-offs between endpoints. From a patients’ perspective, endpoints should not be viewed in isolation but evaluated as a ‘combined package’ – e.g. ‘survival in the best possible vs worst possible wellbeing state’, or ‘time spent without progression and toxicity’ would be more practical measures to patients (i.e. overall health/QoL vs time). PRO and QoL data can put new treatment benefits in context and enable more useful comparisons between available treatment options. However, with a lack of evidential standards for health-related QoL in clinical oncology, current PRO tools are sometimes not fit for purpose. Consequently, with patient experience not being captured in a meaningful way, crucial data is missing from the benefit-risk assessment, and treatment decisions are being made with an incomplete picture.

In order to rectify the status quo, patient preference data must be collected in a standardized and systematic manner to translate patients’ perspectives from subjective to quantifiable, reliable and robust outcomes. Current PRO collection/reporting lacks granularity and specificity, with QoL often being presented in vague terms, as one or few non-descript outcomes, rather than providing any tangible treatment effect on aspects fundamental to daily life such as: independence/autonomy (e.g. need for a carer); cognitive function (e.g. memory loss); differentiation between age groups or other subpopulations (e.g. living with peripheral neuropathy in your 30s–50s versus in your 80s makes a difference in terms of gait and grip). Thus, QoL is a critical component of treatment effectiveness which can severely impact overall wellbeing, yet is often inadequately addressed in clinical trial designs.

Consequently, there is an urgent need for QoL and patient preferences to comprise more than just a ‘ticked box’ in clinical cancer research, and for PROs to transform from weak qualitative information (often reported as an after-thought) into objective outcomes on par with other clinical outcomes. Finally, there must be greater recognition across all stakeholders—regulators, HTA bodies, sponsors, and clinicians—of the real trade-offs patients themselves are willing to make between outcomes. This requires more transparent communication about benefit–risk uncertainties and a commitment to patient-informed evidence generation.

## Marketing Approval Does Not Mean Market (Nor Patient) Access

1. HTA and reimbursement considerations

Healthcare policy goals in Europe consist in ensuring affordable and equitable access to effective therapies for all patients in a sustainable manner, and regulatory approval is just a step on the road to patient access. Once a particular precision oncology drug receives EU MA, access may be delayed by a number of hurdles. One problem is the consistent rise in healthcare expenditures across all EU countries over the last two decades, mainly attributed to improved life expectancy and comorbidities linked to ageing [44]. Alongside this factor is the advent of new technologies, with the cost of drugs rising exponentially over the years. For example, in Europe, expenditures on cancer drugs more than tripled from $10 billion in 2005 to $32 billion in 2018 [45]. Global healthcare costs are set to rise in the coming years, with predicted cancer cases expected to increase worldwide [46]. Rising costs aside, other factors considered by HTA and payers are the potential logistical and infrastructural challenges to routinely implement effective diagnostics and deploy treatments in already stretched healthcare systems, such as site heterogeneity, assay turnaround, MRD integration, preference elicitation burden, and the necessary resources (staff, expertise, capacity).

**Fig. 4:**
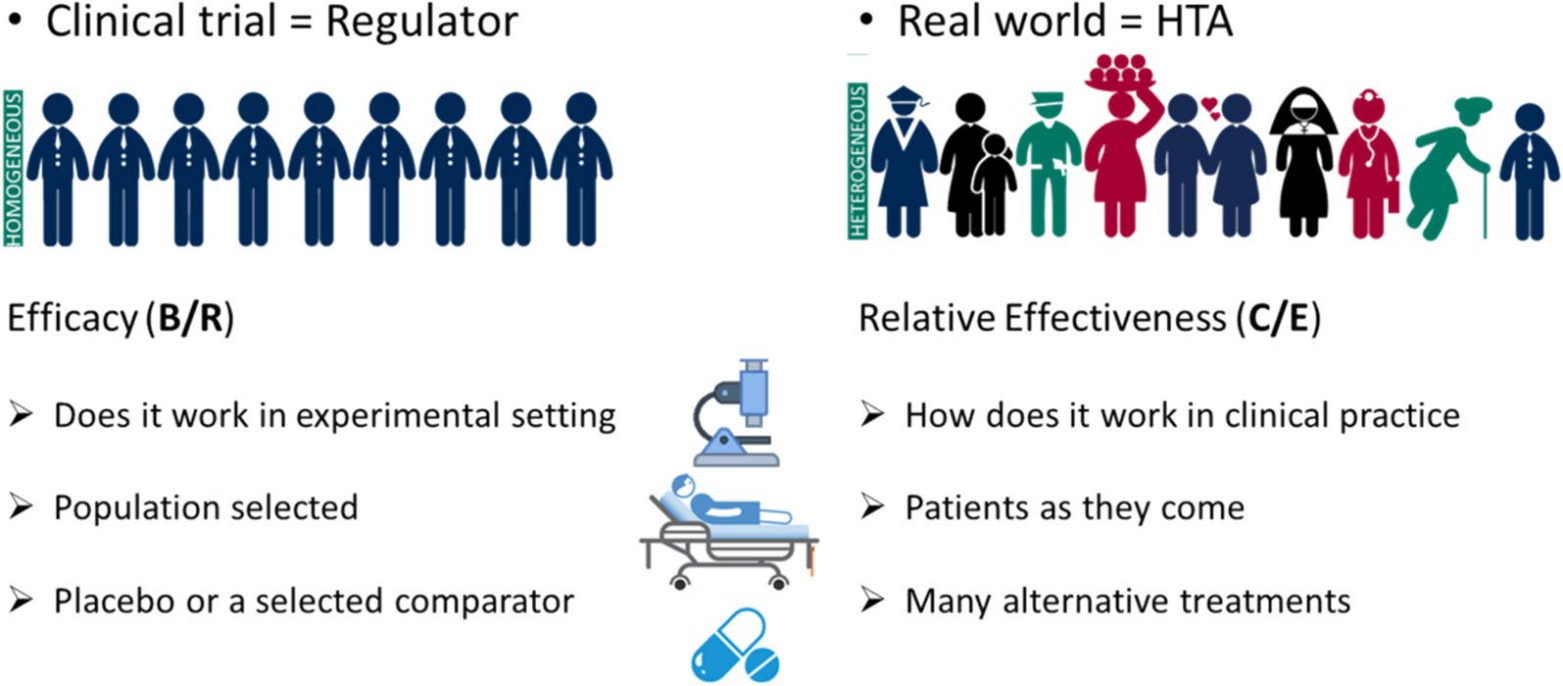
Approval vs access. Based on the presentation at the conference by Anja Schiel (Norwegian Medical Products Agency [NOMA], Norway). B/R, benefit-risk; C/E, cost-effectiveness; HTA, health technology assessment

HTA is a multidisciplinary methodological process determining the value of a given new therapy to inform decision-making, in order to promote an equitable, efficient and high-quality national health system. HTA agencies across individual EU countries assess whether a new drug should be reimbursed by examining overall cost-benefit to society. While clinical trials assess treatment efficacy (benefit-risk) in a highly select group of participants, HTA evaluates relative effectiveness for patients in the real world while also assessing treatment cost/expense to individual healthcare systems (Figure 4). Therefore, HTA is not merely about appraising efficacy and safety of a new drug, rather it contextualizes its added-value by asking the question of internal vs external validity (i.e., truth in a study vs truth in real life – ‘just because a drug works does not mean a government should buy it’).

In order to ascertain the added-value of a new drug, HTA requires an anchor to compare relative effectiveness vs other new/existing health technologies. This is particularly important when new drugs are being developed for a treatment setting for which there are already available treatment alternatives. From an HTA perspective, randomization between a new intervention and best practice is essential for the interpretation of data and for making sound decisions, and an RCT in a small population provides more robust data than an SAT with a larger population – the only real exception for not having a robust comparator is in ultra-rare cancers. HTA does not necessarily require statistical significance, as long as subgroups have been pre-defined from the start and a clinically meaningful treatment effect can be demonstrated. Finally, relative effectiveness is also at its core a question about diversity which is also an important factor for reimbursement decisions. Every country’s healthcare ministry is responsible for managing a set budget and for spending as little as possible (i.e., paying for something new will inevitably take money away from something/someone else), which can often clash with industry’s business plans. There is a greater need for honest but often uncomfortable conversations about the societal/financial trade-offs in HTA decision-making.

(2) EU HTAR and patient involvement in HTA

Drug affordability differs significantly across EU countries, largely influenced by variations in gross domestic product. Consequently, EU HTA agencies remain highly heterogeneous in their criteria for reimbursement (none of the ~50 HTA agencies across the 27 EU states are identical) [47]. Despite divergent national healthcare budgets and policy priorities, HTA bodies should strive for collective alignment on their data requirements to streamline drug development and ultimately facilitate equitable patient access. This notion led to the HTAR (Regulation 2021/2282), effective since January 2025 to promote a more uniform decision-making basis for medical technology assessments throughout the EU, by establishing a framework for joint clinical assessments (JCA) at the EU level incorporating close collaboration with the EMA and patient involvement [6]. HTAR also established joint scientific consultations (JSC) at EU level enabling scientific consultation by industry during their clinical development planning regarding evidence needs for a subsequent JCA. These initiatives should encourage efficient EMA and HTA decision-making and be a positive step forward to avoid unnecessary redundancies for industry.

Traditionally, HTA processes have relied heavily on clinical and economic data, which often fail to reflect patient-relevant outcomes and the patient experience (e.g. treatment tolerability, QoL, burden of symptoms and AEs, psychological impact). Under HTAR, patient involvement is encouraged (not mandated) at JCAs and JSCs, offering a valuable opportunity to move from tokenistic engagement to a more systematic and impactful integration of PED and patient preference studies. It also begins to recognize the economic and clinical value of patients’ insights. Without meaningful patient input in HTA, we risk reimbursing on the public purse therapies that fail to meet patients’ needs (i.e. why have a new treatment that patients do not wish to take), or rejecting treatments that may in fact significantly improve daily life. As such, involving patients is not just a moral imperative – it is an evidence and value imperative.

It is noteworthy, however, that this new legislation is more intentional than fully binding. Although patient involvement is increasing, major barriers remain including lack of consistency and strategy on how to integrate patient data into the decision-making process (i.e. lack of guidelines), tokenistic engagement, resistance from some stakeholders to fully embrace shared decision-making, process complexity and administrative burden, and limited training and capacity-building for the participants. Therefore, HTAR raises high expectations on harmonization, faster access and integrating the patient voice, but it remains to be seen whether it will effectively address long-standing inequities in access across Europe. Its success will depend on transparent processes, clearly defined evidence standards, and genuine inclusion of patient perspectives – not only as contributors, but as co-creators of value.

## Conclusions

Despite leaps in progress in cancer treatment, patient access to effective innovative therapies remains slow and highly inconsistent across the EU. Smarter randomized study designs (even with small numbers), pragmatic or mixed design trials, and validated prospectively-planned ECAs (e.g. in ultra-rare diseases) could generate more robust evidence from the get-go, for swifter regulatory approval and HTA procedures, ultimately streamlining patient access. The evidence-based development of new, more reliable and timely endpoints will be a key challenge that can be met by close collaboration between multiple pharmaceutical companies, commercial laboratories, academic researchers, patient advocates and regulators. There is a growing recognition of the importance of patient preference studies in clinical cancer research and the need for systematic, standardized collection of PROs to generate tangible QoL data for consideration alongside efficacy and safety in regulatory and HTA decision-making. Furthermore, adequate representation of relevant populations in clinical research goes beyond concern for social justice, and is firstly a public health issue with strong scientific and business backing. Therefore, smarter study designs should also include patient consultation from the earliest planning stages and broader trial participant representation reflecting the real-world target population.

Panel: Call to Action to Generate Robust Data that will Facilitate Patient Access to (Precision) Oncology Therapies:

Smarter Trial Designs in Clinical Cancer Research:

- All-stakeholder collaboration to advance innovative methods of evidence generation that produce robust data meeting the evidence requirements for regulatory and health technology assessment (HTA) decision-makers (e.g., pragmatic trials, mixed trial/partially randomized patient preference designs) in order to expedite access to novel cancer therapies.
  o Regulators and payers require the highest level of data robustness, especially with increasing treatment options available in a given setting – only randomization (even with small sample size, not necessarily powered to detect differences in time-to-event endpoints) can demonstrate relative effectiveness and improve decision-making in clinical development. Innovative trial designs should be explored (e.g., borrowing data or augmenting control arms).
  o When randomization is really not feasible (e.g., for some ultra-rare cancers or in the absence of an effective treatment), the value of external control data should be increased through prospectively designed trials. Early prospective planning is fundamental for the use of an external control arm (if needed), and collection of the necessary data should be done concurrently alongside the initial stages of trial design, rather than as an after-thought at data analysis or submission stages – a sub-optimally designed trial cannot be ‘rescued’ by unplanned, retrospectively-collected external data.
  o “Every patient counts” – we must find ways to collect robust data from patients in the broader population and not only those who are eligible and have the possibility to participate in clinical trials. If we ask drug developers to prospectively design their controls, they will require an adequate pool to source these; as a community, we must enable this by proactively seeking patients’ consent for data/tissue samples/procedures.
  o Patients should be involved early on in the trial design process, from the inception of the first protocol and throughout the drug development process, to conduct more relevant and impactful trials.

New Endpoints in Clinical Cancer Research

- Foster multi-stakeholder collaboration and sharing of data for the development and validation of new endpoints – be it surrogate or earlier, intermediate efficacy endpoints or standardized quality of life (QoL), and more systematic use of validated patient-reported outcome (PRO) measures.
  o *Surrogate/intermediary endpoints*: Earlier read-outs that inform on (presence/lack of) treatment efficacy in a specific population within a trial would enable patients to benefit from an alternative dosing or treatment as soon as possible and also facilitate the design of subsequent strategies (e.g., other areas of unmet need or to prioritize those patient populations in which a certain regimen may provide substantial benefit and for whom more information is needed).
  o *Standardizing patient-reported outcomes*: Smarter trial designs should systematically capture patient preferences, PROs and QoL data in a standardized manner to capture patients’ experience beyond their clinical outcomes (https://www.sisaqol-imi.org/).
  o A collective effort is required to advance the development, standardization and validation of relevant PROs in cancer in order to inform clinical development, but also beyond drug approval for HTA decision-making on reimbursement and treatment optimization in clinical use.
  o Patients’ perspectives should be considered in decision-making on treatment choices and reimbursement, therefore regulators and HTA agencies must lead the way in requiring that PROs including QoL measures be integrated into clinical trial designs at the same level of importance/priority as efficacy and safety outcome measures.

Representative Patient Inclusion in Clinical Cancer Research

- A concerted effort among all stakeholders is required to increase patient representation in oncology clinical trials to achieve an actual increase in diversity reflecting real-world epidemiology.
  o Strive for better representation not only to ensure generalizability and adequate efficacy and safety assessments for all populations, but also to ensure equal access. This is a necessary pillar for achieving healthcare for all.
  o Ensure that poorly represented groups are included in the planning, conduct, analysis and evaluation of clinical trials through increased knowledge about target populations and adequate inclusion/exclusion criteria in clinical trials.
  o Envisage how clinical trials can be planned and analyzed to ensure that the needs of all patient groups can be addressed, e.g. through pre-planned data collection and disaggregated analyses of relevant groups (including by sex and/or age groups, depending on the setting).

## Data Availability

No datasets were generated or analysed during the current study.

## References

[1] European Parliamentary Research Service (EPRS). European pharmaceutical research and development – Could public infrastructure overcome market failures? European Union, December 2021. https://www.europarl.europa.eu/RegData/etudes/STUD/2021/697197/EPRS_STU(2021)697197_EN.pdf

[2] Uyl-de Groot CA, Heine R, Krol M, et al. Unequal access to newly registered cancer drugs leads to potential loss of life-years in Europe. Cancers. 2020;12(8):2313. 32824444 10.3390/cancers12082313 PMC7464890

[3] Post HC, Schutte T, van Oijenet MGH, et al. Time to reimbursement of novel anticancer drugs in Europe: a case study of seven European countries. ESMO Open. 2023;8(2):101208. 37030113 10.1016/j.esmoop.2023.101208 PMC10163159

[4] Lawler M, Bhatti S, Przewiezlikowski P, et al. Central and Eastern Europe: A crucible for clinical cancer innovation? Journal of Cancer Policy 2025; 46:100647. 10.1016/j.jcpo.2025.100647 41106494

[5] European Commission. COMBINE programme for clinical trials and medical devices endorsed by Member States, December 2024. https://ec.europa.eu/newsroom/sante/items/862110/en

[6] European Commission. New EU rules on Health Technology Assessment open up a new era for patient access to innovation, 10 January 2025. https://ec.europa.eu/commission/presscorner/detail/en/ip_25_226

[7] Mulder J, Teerenstra S, van Hennik PB, et al. Single-arm trials supporting the approval of anticancer medicinal products in the European Union: contextualization of trial results and observed clinical benefit. ESMO Open. 2023;8(2):101209. 37054504 10.1016/j.esmoop.2023.101209 PMC10163162

[8] Agrawal S, Arora S, Amiri-Kordestani L, et al. Use of single-arm trials for US Food and Drug Administration drug approval in oncology, 2002–2021. JAMA Oncol. 2023;9(2):266–72. 36580315 10.1001/jamaoncol.2022.5985

[9] Wang M, Ma H, Shi Y, et al. Single-arm clinical trials: design, ethics, principles. BMJ Support Palliat Care. 2024;15(1):46–54. 38834238 10.1136/spcare-2024-004984 PMC11874317

[10] Burger HU, Gerlinger C, Harbron C, et al. The use of external controls: to what extent can it currently be recommended? Pharm Stat. 2021;20(6):1002–16. 33908160 10.1002/pst.2120

[11] Billigham L, Brown L, Framke T, et al. Histology independent drug development - Is this the future for cancer drugs? Cancer Treat Rev. 2024;123:102674. 38176220 10.1016/j.ctrv.2023.102674

[12] Lewis CJ, Sarkar S, Zhu J, et al. Borrowing from historical control data in cancer drug development: a cautionary tale and practical guidelines. Stat Biopharm Res. 2019;11(1):67–78. 31435458 10.1080/19466315.2018.1497533 PMC6703839

[13] Edwards D, Best N, Crawford J, et al. Using Bayesian dynamic borrowing to maximize the use of existing data: a case-study. Ther Innov Regul Sci. 2024;58(1):1–10. 37910271 10.1007/s43441-023-00585-3 PMC10764450

[14] Kessels R, May AM, Koopman M, et al. The trial within cohorts (TwiCs) study design in oncology: experience and methodological reflections. BMC Med Res Methodol. 2023. 10.1186/s12874-023-01941-5. 37179306 10.1186/s12874-023-01941-5 PMC10183126

[15] Relton C, Torgerson D, O’Cathain A, et al. Rethinking pragmatic randomised controlled trials: introducing the “cohort multiple randomised controlled trial” design. BMJ. 2010;340:c1066. 20304934 10.1136/bmj.c1066

[16] Wasmann KA, Wijsman P, van Dieren S, et al. Partially randomised patient preference trials as an alternative design to randomised controlled trials: systematic review and meta-analyses. BMJ Open. 2019;9(10):e031151. 31619428 10.1136/bmjopen-2019-031151 PMC6797441

[17] Bach SP. STAR-TREC: an international three-arm multicentre, partially randomised controlled trial incorporating an external pilot. Clin Oncol (R Coll Radiol). 2023;35(2):e107–9. 36577551 10.1016/j.clon.2022.12.006

[18] Wang Y, Li F, Blaha O, et al. Design and analysis of partially randomized preference trials with propensity score stratification. Stat Methods Med Res. 2022;31(8):1515–37. 35469503 10.1177/09622802221095673 PMC10530658

[19] Alsop J, Scott M, Archey W. The mixed randomized trial: combining randomized, pragmatic and observational clinical trial designs. J Comp Eff Res. 2016;5(6):569–79. 27618500 10.2217/cer-2016-0034

[20] Liu R, Rizzo S, Whipple S, et al. Evaluating eligibility criteria of oncology trials using real-world data and AI. Nature. 2021;592:629–33. 33828294 10.1038/s41586-021-03430-5 PMC9007176

[21] Shenoy P, Harugeri A. Elderly patients’ participation in clinical trials. Perspect Clin Res. 2015;6(4):184–9. 26623388 10.4103/2229-3485.167099 PMC4640010

[22] De Rojas T, Neven A, Terada M, et al. Access to clinical trials for adolescents and young adults with cancer: a meta-research analysis. JNCI Cancer Spectr. 2019;3(4):pkz057. 32337483 10.1093/jncics/pkz057 PMC7050014

[23] Gross AS, Harry AC, Clifton CS, et al. Clinical trial diversity: an opportunity for improved insight into the determinants of variability in drug response. Br J Clin Pharmacol. 2022;88(6):2700–17. 35088432 10.1111/bcp.15242 PMC9306578

[24] World Medical Association, WMA International Code of Medical Ethics, April 2023. https://www.wma.net/policies-post/wma-international-code-of-medical-ethics/

[25] Versavel S, Subasinghe A, Johnson K, et al. Diversity, equity, and inclusion in clinical trials: a practical guide from the perspective of a trial sponsor. Contemp Clin Trials. 2023;126:107092. 36702295 10.1016/j.cct.2023.107092

[26] Delgado A, Guddati AK. Clinical endpoints in oncology – a primer. Am J Cancer Res. 2021;11(4):1121–31. 33948349 PMC8085844

[27] Cooper K, Tappenden P, Cantrell A, et al. A systematic review of meta-analyses assessing the validity of tumour response endpoints as surrogates for progression-free or overall survival in cancer. Br J Cancer. 2020;123:1686–96. 32913287 10.1038/s41416-020-01050-w PMC7687906

[28] Booth CM, Eisenhauer EA, Gyawali B, et al. Progression-free survival should not be used as a primary end point for registration of anticancer drugs. J Clin Oncol. 2023;41:4968–72. 37733981 10.1200/JCO.23.01423

[29] Falcone R, Lombardi P, Filetti M, et al. Oncologic drugs approval in Europe for solid tumors: overview of the last 6 years. Cancers. 2022;14(4):889. 35205637 10.3390/cancers14040889 PMC8870299

[30] Durie B. A historic turning point: ODAC unanimously votes in favor of MRD testing as an early endpoint in myeloma clinical trials to support accelerated approvals of new treatments. International Myeloma Foundation; April 2024. https://www.myeloma.org/blog/dr-duries/odac-unanimously-in-favor-mrd-testing-early-endpoint-myeloma

[31] Ailawadhi S, Arnulf B, Patel K, et al. Ide-cel vs standard regimens in triple-class-exposed relapsed and refractory multiple myeloma: updated KarMMa-3 analyses. Blood. 2024;144(23):2389–401. 39197072 10.1182/blood.2024024582

[32] Sonneveld P, Dimopoulos MA, Boccadoro M, et al. Daratumumab, bortezomib, lenalidomide, and dexamethasone for multiple myeloma. N Engl J Med. 2024;390(4):301–13. 38084760 10.1056/NEJMoa2312054

[33] Facon T, Kumar SK, Plesner T, et al. Daratumumab, lenalidomide, and dexamethasone versus lenalidomide and dexamethasone alone in newly diagnosed multiple myeloma (MAIA): Overall survival results from a randomised, open-label, phase 3 trial. Lancet Oncol. 2021;22(11):1582–96. 34655533 10.1016/S1470-2045(21)00466-6

[34] Mai EK, Bertsch U, Pozek E, et al. Isatuximab, lenalidomide, bortezomib, and bexamethasone induction therapy for transplant-eligible newly diagnosed multiple myeloma: Final part 1 analysis of the GMMG-HD7 trial. J Clin Oncol 2024:JCO2402266. 10.1200/JCO-24-02266 PMC11974620 39652594

[35] Moreau P, Dimopoulos M, Mikhael J, et al. Isatuximab, carfilzomib, and dexamethasone in relapsed multiple myeloma (IKEMA): a multicentre, open-label, randomised phase 3 trial. Lancet. 2021;397(10292):2361–71. 34097854 10.1016/S0140-6736(21)00592-4

[36] Zabaleta A, Puig N, Cedena M, et al. Clinical significance of complete remission and measurable residual disease in relapsed/refractory multiple myeloma patients treated with T-cell redirecting immunotherapy. Am J Hematol. 2025;100(1):93–102. 39548827 10.1002/ajh.27526 PMC11625977

[37] Paiva B, Zherniakova A, Nuñez-Córdoba JM, et al. Impact of treatment effect on MRD and PFS: An aggregate data analysis from randomized clinical trials in multiple myeloma. Blood Adv. 2024;8(1):219–23. 37639322 10.1182/bloodadvances.2023010821 PMC10805640

[38] Shi Q, Paiva B, Pederson LD, et al. Minimal residual disease-based end point for accelerated assessment of clinical trials in multiple myeloma: a pooled analysis of individual patient data from multiple randomized trials. J Clin Oncol. 2025;43(11):1289–301. 39938021 10.1200/JCO-24-02020

[39] Boyiadzis M, Wei AH, Paiva B, et al. Measurable residual disease (MRD) as a surrogate end point for clinical drug approval in acute myeloid leukemia (AML): perspectives from the MRD partnership and alliance in AML clinical treatment consortium. Cancer. 2025;131(13):e35960. 40576165 10.1002/cncr.35960 PMC12203635

[40] Tettero J, Eric S, Freeman S, et al. Validation of measurable residual disease as a surrogate endpoint in acute myeloid leukemia: a HARMONY Alliance study of European randomized trials. Blood. 2025;146(Suppl 1):343.

[41] Landgren O, Devlin SM. Minimal residual disease as an early endpoint for accelerated drug approval in myeloma: a roadmap. Blood Cancer Discov. 2025;6(1):13–22. 39630969 10.1158/2643-3230.BCD-24-0292 PMC11707509

[42] Powles T, Kann AG, Castellano D, et al. ctDNA-guided adjuvant atezolizumab in muscle-invasive bladder cancer. N Engl J Med. 2025;393(24):2395–408. 41124204 10.1056/NEJMoa2511885

[43] Pe M, Alanya A, Falk RS, et al. Setting international standards in analyzing patient-reported outcomes and quality of life endpoints in cancer clinical trials-innovative medicines initiative (SISAQOL-IMI): stakeholder views, objectives, and procedures. Lancet Oncol. 2023;24(6):e270–83. 37269858 10.1016/S1470-2045(23)00157-2

[44] Eurostat, Healthcare expenditure statistics – overview, November 2025. https://ec.europa.eu/eurostat/statistics-explained/index.php?title=Healthcare_expenditure_statistics_-_overview

[45] Hofmarcher T, Lindgren P, Wilking N, et al. The cost of cancer in Europe 2018. Eur J Cancer. 2020;129:41–9. 32120274 10.1016/j.ejca.2020.01.011

[46] WHO (World Health Organisation), Global cancer burden growing, amidst mounting need for services, February 2024. https://www.who.int/news/item/01-02-2024-global-cancer-burden-growing--amidst-mounting-need-for-services PMC11115397 38438207

[47] European Commission. Mapping of HTA national organisations, programmes and processes in EU and Norway, May 2017. https://health.ec.europa.eu/document/download/18a3dc1d-4876-4553-a292-84fc4a7f9e31_en

[48] Paiva B, Shi Q, Puig N, et al. Opportunities and challenges for MRD assessment in the clinical management of multiple myeloma. Nat Rev Clin Oncol. 2025;22(6):424–38. 40195455 10.1038/s41571-025-01017-x

[49] Heinrich K, Modest DP, Ricard I, et al. Gender-dependent survival benefit from first-line irinotecan in metastatic colorectal cancer. Subgroup analysis of a phase III trial (XELAVIRI-study, AIO-KRK-0110). Eur J Cancer. 2021;147:128–39. 33647548 10.1016/j.ejca.2021.01.025

[50] Margalit O, Harmsen WS, Shacham-Shmueli E, et al. Evaluating sex as a predictive marker for response to bevacizumab in metastatic colorectal carcinoma: pooled analysis of 3,369 patients in the ARCAD database. Eur J Cancer. 2023;178:162–70. 36446161 10.1016/j.ejca.2022.10.022

[51] Wagner AD, Grothey A, Andre T, et al. Sex and adverse events of adjuvant chemotherapy in colon cancer: an analysis of 34 640 patients in the ACCENT database. J Natl Cancer Inst. 2021;113(4):400–7. 32835356 10.1093/jnci/djaa124 PMC8023830

[52] Brioli A, Nägler TM, Yomade O, et al. Sex-disaggregated analysis of biology, treatment tolerability, and outcome of multiple myeloma in a German cohort. Oncol Res Treat. 2022;45(9):494–503. 35705004 10.1159/000525493

[53] Blijlevens N, Schwenkglenks M, Bacon M, et al. Prospective oral mucositis audit: oral mucositis in patients receiving high-dose melphalan or BEAM conditioning chemotherapy – European Blood and Marrow Transplantation Mucositis Advisory Group. J Clin Oncol. 2008;26(9):1519–25. 18268357 10.1200/JCO.2007.13.6028

[54] Ozga M, Nicolet D, Mrózek K, et al. Sex-associated differences in frequencies and prognostic impact of recurrent genetic alterations in adult acute myeloid leukemia (Alliance, AMLCG). Leukemia. 2024;38(1):45–57. 38017103 10.1038/s41375-023-02068-8 PMC10776397

[55] GenoMed4All Consortium. A sex-informed approach to improve the personalised decision making process in myelodysplastic syndromes: a multicentre, observational cohort study. Lancet Haematol. 2023;10(2):e117–28. 36436542 10.1016/S2352-3026(22)00323-4 PMC9886555

[56] Torres JM, Sodipo MO, Hopkins MF, et al. Racial differences in breast cancer survival between black and white women according to tumor subtype: a systematic review and meta-analysis. J Clin Oncol. 2024;42:3867–79. 39288352 10.1200/JCO.23.02311 PMC11540747

[57] Merz LE, Story CM, Osei MA, et al. Absolute neutrophil count by Duffy status among healthy Black and African American adults. Blood Adv. 2023;7:317. 35994632 10.1182/bloodadvances.2022007679 PMC9881043

[58] Merz LE, Osei MA, Stor CM, et al. Development of Duffy null–specific absolute neutrophil count reference ranges. JAMA. 2023;329(23):2088–9. 37338884 10.1001/jama.2023.7467 PMC10282887

[59] Hershman D, Weinberg M, Rosner Z, et al. Ethnic neutropenia and treatment delay in African American women undergoing chemotherapy for early-stage breast cancer. J Natl Cancer Inst. 2003;95(20):1545–8. 14559877 10.1093/jnci/djg073

